# High-Quality Genome-Scale Reconstruction of *Corynebacterium glutamicum* ATCC 13032

**DOI:** 10.3389/fmicb.2021.750206

**Published:** 2021-11-15

**Authors:** Martina Feierabend, Alina Renz, Elisabeth Zelle, Katharina Nöh, Wolfgang Wiechert, Andreas Dräger

**Affiliations:** ^1^Computational Systems Biology of Infections and Antimicrobial-Resistant Pathogens, Institute for Bioinformatics and Medical Informatics (IBMI), University of Tübingen, Tübingen, Germany; ^2^Department of Computer Science, University of Tübingen, Tübingen, Germany; ^3^Institute of Bio- and Geosciences, IBG-1: Biotechnology, Forschungszentrum Jülich GmbH, Jülich, Germany; ^4^Computational Systems Biotechnology (AVT.CSB), RWTH Aachen University, Aachen, Germany

**Keywords:** *Corynebacterium glutamicum*, genome-scale metabolic model, constraint-based reconstruction, optimization, metabolic engineering, FAIR, flux balance analysis, MEMOTE

## Abstract

*Corynebacterium glutamicum* belongs to the microbes of enormous biotechnological relevance. In particular, its strain ATCC 13032 is a widely used producer of L-amino acids at an industrial scale. Its apparent robustness also turns it into a favorable platform host for a wide range of further compounds, mainly because of emerging bio-based economies. A deep understanding of the biochemical processes in *C. glutamicum* is essential for a sustainable enhancement of the microbe's productivity. Computational systems biology has the potential to provide a valuable basis for driving metabolic engineering and biotechnological advances, such as increased yields of healthy producer strains based on genome-scale metabolic models (GEMs). Advanced reconstruction pipelines are now available that facilitate the reconstruction of GEMs and support their manual curation. This article presents *i*CGB21FR, an updated and unified GEM of *C. glutamicum* ATCC 13032 with high quality regarding comprehensiveness and data standards, built with the latest modeling techniques and advanced reconstruction pipelines. It comprises 1042 metabolites, 1539 reactions, and 805 genes with detailed annotations and database cross-references. The model validation took place using different media and resulted in realistic growth rate predictions under aerobic and anaerobic conditions. The new GEM produces all canonical amino acids, and its phenotypic predictions are consistent with laboratory data. The *in silico* model proved fruitful in adding knowledge to the metabolism of *C. glutamicum*: *i*CGB21FR still produces L-glutamate with the knock-out of the enzyme pyruvate carboxylase, despite the common belief to be relevant for the amino acid's production. We conclude that integrating high standards into the reconstruction of GEMs facilitates replicating validated knowledge, closing knowledge gaps, and making it a useful basis for metabolic engineering. The model is freely available from BioModels Database under identifier MODEL2102050001.

## 1. Introduction

The strain *Corynebacterium glutamicum* ATCC 13032 is a Gram-positive, facultatively anaerobic soil bacterium, which produces L-glutamate under particular treatments or growth conditions (Kimura, 2005). The annual production of several tons of L-glutamate (Eggeling and Bott, 2005) as well as other metabolically engineered products, such as other amino acids (Eggeling and Bott, 2015; Wendisch et al., 2016), alcohols (Inui et al., 2004a; Niimi et al., 2011; Yamamoto et al., 2013; Jojima et al., 2015), biopolymers (Liu et al., 2007), organic acids (Hüser et al., 2005; Okino et al., 2008; Takeno et al., 2013), terpenoids (Heider et al., 2014; Kang et al., 2014) or diamines (Kind et al., 2010a,b; Schneider and Wendisch, 2010), have turned *C. glutamicum* into a versatile and enormously relevant biotechnological microorganism. Despite an ongoing biotechnological application of *C. glutamicum* and the resulting knowledge on this bacterium for more than 70 years (Vertes et al., 2013), its metabolic potential not yet exhausted. Due to the prominent role of *C. glutamicum* in biotechnology, obtaining a more profound understanding of its physiology and metabolism is highly desirable.

One method of formalizing this knowledge is a genome-scale metabolic network reconstruction. Genome-scale metabolic network reconstructions represent a systematic knowledge base of bibliomic and genomic data of all known metabolic reactions of a specific target organism (Thiele and Palsson, 2010). By creating a mathematical representation of the reconstructed network, the network can be changed into a genome-scale metabolic model (GEM). GEMs enable the qualitative description of the genotype-phenotype relationship and predictions of various phenotypes (Fang et al., 2020).

GEMs can be constructed by mapping the annotated genome sequence with its genes via the encoded proteins to reactions. This step is followed by an intensive curation phase of the computational model and a subsequent analysis phase. Prevalent methods for analyzing GEMs are summarized under the therm constraint-based modeling. The main advantage of these modeling techniques over other approaches, such as dynamic modeling (Dräger et al., 2009), lies in their potential to analyze entire metabolic networks at the scale of all enzymatic capabilities of an organism without the necessity of knowing numerical values of all the kinetic parameters therein. Flux sampling can be used as an unbiased way to characterize the space of stoichiometrically feasible fluxes and solutions (Jadebeck et al., 2020). Flux balance analysis (FBA) is a biased method for steady-state analysis of GEMs. By imposing further physiologically realistic, relevant constraints and a target objective function on the computational model, the network's metabolic flux distributions can be simulated (Fang et al., 2020). Nevertheless, increasing network scale results in an increasingly complex process of reconstructing all cellular properties in the form of a coherent computer model.

**Figure 1:**
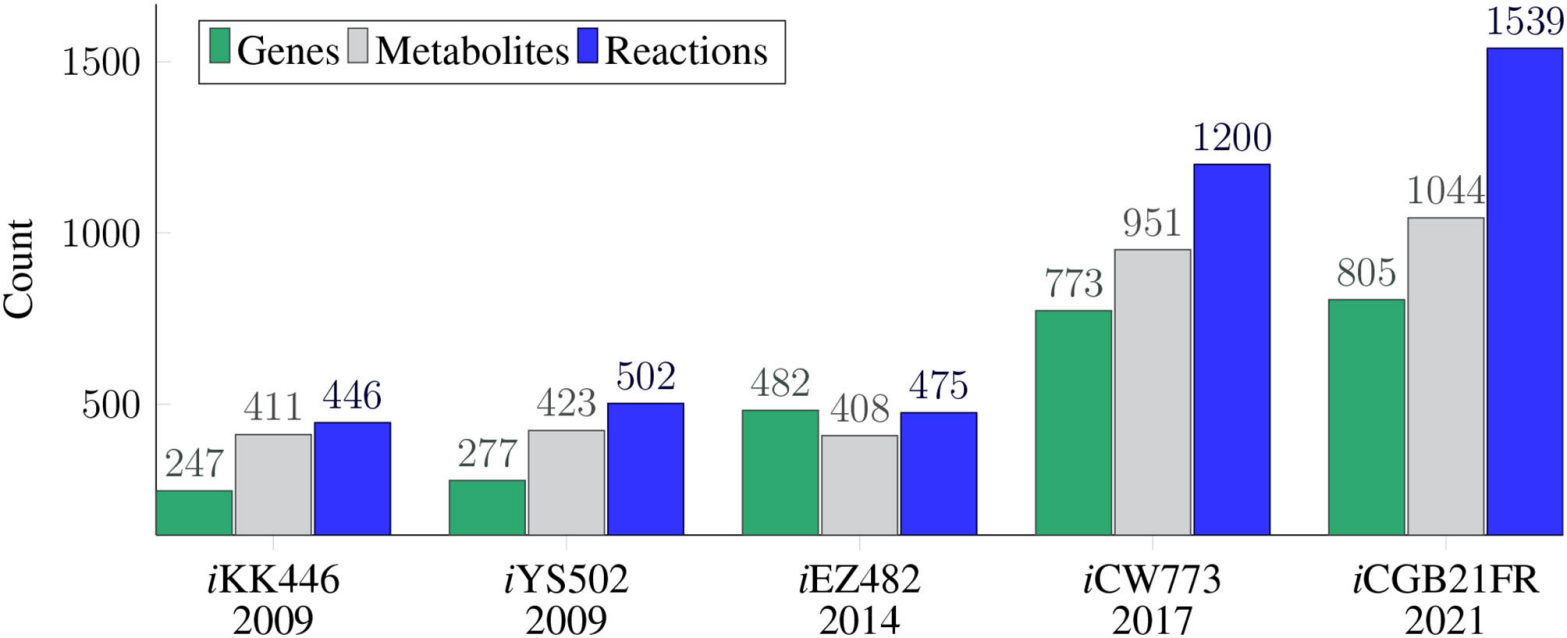
Timeline and model history of all available *C. glutamicum* genome-scale metabolic models. The GEMs are depicted in the chronological order of their publication dates, with *i*KK446 as the first available GEM of *C. glutamicum*. The figure elucidates which GEM is based on which previous GEMs. The upper part of the timeline depicts the number of reactions (blue), metabolites (gray), and genes (green) for each of the five GEMs of *C. glutamicum*. While the number of reactions, metabolites, and genes in the first three GEMs are comparable in their magnitude, the number of metabolites and genes more than doubled in the most recent two GEMs. The number of reactions more than doubled in *i*CW773 (Zhang et al., 2017) and more than tripled in *i*CGB21FR. The model *i*CGB21FR is an updated GEM of *i*EZ482 based on the first published GEM of *C. glutamicum i*KK446. The model *i*CW773 is based on the shortly later published GEM *i*YS502.

In recent years, new tools and automated techniques in systems biology have emerged, such as CarveMe (Machado et al., 2018), ModelPolisher (Römer et al., 2016), Memote (Lieven et al., 2020), or BOFdat (Lachance et al., 2019). These tools support the reconstruction, refinement, and validation of GEMs using Minimal Information Required In the Annotation of Models (MIRIAM) standards (Le Novère et al., 2005). Several GEMs of the *C. glutamicum* have already been published (e.g., Kjeldsen and Nielsen, 2009; Shinfuku et al., 2009, see Figure 1). However, these models were curated before the newly developed tools were available. Thus, these new tools were so far not applied to GEMs of *C. glutamicum*. The most recently published GEM of *C. glutamicum* is *i*CW773 (Zhang et al., 2017), which is based on Shinfuku et al. (2009). The model *i*CW773 can produce all canonical amino acids. The production rates of amino acids are generally lower than experimental results (Eggeling and Bott, 2005). Comparing these production rates to those of other published GEMs of *C. glutamicum* is difficult since neither the composition of the complete medium nor the medium used for the *in silico* experiments is reported. Based on the Memote report of *i*CW773, the model seems to lack stochiometric consistency and contains no Systems Biology Ontology (SBO) terms (see below for more information on SBO terms Courtot et al., 2011). It contains 98 orphan and 116 dead-end metabolites. In the respiratory chain, the metabolites ubiquinone and its derivates are used. However, several experimental studies confirmed that the only respiratory quinones in *C. glutamicum* are menaquinone and its derivates (Kanzaki et al., 1974; Collins et al., 1977, 1979; Bott and Niebisch, 2003; Maeda et al., 2020). After conversion to Systems Biology Markup Language (SBML) Level 3 Version 1 (Hucka et al., 2018), *i*CW773 reaches a total Memote score of only 29 % (Lieven et al., 2020, see below for more information on Memote and this score). Newly available tools such as Memote have not yet been applied to reconstruct any previous GEM of *C. glutamicum*. The goal of this model is to fill this application gap. Given its importance as a biotechnological microbe, an updated GEM reflecting the current state of knowledge about *C. glutamicum* and incorporating the scope of newly available tools is indispensable.

In this study, we present an updated GEM of high quality for *C. glutamicum* named *i*CGB21FR. It combines the knowledge about *C. glutamicum* from the previous models *i*KK446 (Kjeldsen and Nielsen, 2009) and *i*EZ482 (Zelle et al., 2015) and extends it by including a broader metabolic coverage than previous models. This GEM was reconstructed using the latest available *in silico* methods and tools and represents a model composed of the most current standards in systems biology. Furthermore, this GEM uses current community standards and follows the best-practice recommendations by Carey et al. (2020). High quality in terms of GEM reconstruction encompasses several aspects, such as a fully annotated GEM in terms of metabolites, reactions, and genes with gene-protein-reaction (GPR) associations. In addition, SBO terms (Courtot et al., 2011) are included in the model. These allow a more fine-grained description of the respective compound. With the aid of the high-quality reconstruction of the GEM, we reproduced experimentally validated findings. This model allows a more accurate *in silico* depiction of the genetic makeup of *C. glutamicum*. The new model *i*CGB21FR contributes to filling knowledge gaps in the metabolism of *C. glutamicum* by providing further information on relevant pathways used in the production of L-glutamate. Finally, this model uses FAIR data standards (findable, accessible, interoperable, reusable; Wilkinson et al., 2016). Access to all data and metadata used in this model is provided. A highly detailed annotation level within the model is used, and the reconstruction process is described as transparently as possible (Carey et al., 2020).

## 2. Materials and Methods

### 2.1. The Metabolic Network Reconstruction Process

#### 2.1.1. Strain

The GEM of the strain *Corynebacterium glutamicum* ATCC 13032 was reconstructed using the annotated genome sequence (accession number: NC006958.1), which was downloaded from the National Center for Biotechnology Information (NCBI) at https://www.ncbi.nlm.nih.gov (Agarwala et al., 2018).

#### 2.1.2. Draft Reconstruction

The reconstruction process closely followed the protocol by Thiele and Palsson (2010). In short, an automated draft reconstruction was created using CarveMe (Machado et al., 2018), version 1.2.2, and stored in the SBML Level 3 Version 1 format (Hucka et al., 2018). The SBML Level 3 extension for flux balance constraints (fbc) version 2 by Olivier and Bergmann (2018) was enabled and used under default settings for the draft reconstruction. SBML represents a machine-readable exchange format that allows manipulating computational models of biological processes (Keating et al., 2020; Renz et al., 2020). The fbc plugin enables adding structured, semantic descriptions for domain-specific model components such as charges, annotations, flux bounds, GPR rules, or chemical formulas of metabolites (Lieven et al., 2020). This initial draft contained 1496 reactions, 1030 metabolites, and 782 genes in the three compartments: extracellular, cytosol, and the periplasm.

Further automated and manual refinement of the reconstruction of *C. glutamicum* was performed using libSBML (Bornstein et al., 2008), version 5.18.0, and COBRApy (Ebrahim et al., 2013), version 0.17.1. All simulations were run using the CPLEX optimizer, version 12.10 by IBM (https://www.ibm.com/analytics/cplex-optimizer). Metabolic pathways were visualized using the Escher software (King et al., 2015). To support the display as standardized Process Description (PD) map (Rougny et al., 2019) in Systems Biology Graphical Notation (SBGN) enabled software (Touré et al., 2020), the Escher maps were converted to the SBGN Markup Language (SBGNML) format (Bergmann et al., 2020) using EscherConverter (https://github.com/draeger-lab/EscherConverter).

#### 2.1.3. Annotations

Cross-references of the model's instances to other databases were shifted from the notes to the annotations field. Additional metadata, such as annotations and cross-references, was added using the ModelPolisher (Römer et al., 2016). The model's genes were annotated using the old and new locus tags from NCBI and the NCBI protein identifier. SBO terms (Courtot et al., 2011) further annotate the model's instances. SBO terms represent controlled vocabularies, which provide semantic information about model components. For metabolites and genes, the general SBO-terms for simple chemical (SBO:0000247) and genes (SBO:0000243) were used, respectively. The SBO terms for the reactions were chosen as precisely as possible using a new curation pipeline (Fritze, 2020).

#### 2.1.4. Refinement of Metabolite Attributes

The draft was curated to include the correct positioning of the metabolites' chemical formulas and charges. All charges were obtained, if more than one charge per compound was available, in the Biochemically, Genetically, and Genomically structured (BiGG) Models database (Norsigian et al., 2019). In the following verification step, the most appropriate charge for a given reaction in a specific compartment was manually chosen and added to the model. Dead-end metabolites and orphan metabolites were identified and, when appropriate, removed.

#### 2.1.5. Manual Extension

Intensive manual curation was done using the databases BiGG (Norsigian et al., 2019), MetaCyc (Caspi et al., 2020), BioCyc (Karp et al., 2019), Kyoto Encyclopedia of Genes and Genomes (KEGG) (Kanehisa et al., 2019), and new bibliomic data. This draft was then revised using the *i*EZ482 model (Zelle et al., 2015) as a reference. The model *i*EZ482 is an updated version of the *i*KK446 model (Kjeldsen and Nielsen, 2009) and contains 475 reactions, 408 metabolites, and 482 genes. Reactions, metabolites, and genes present in *i*EZ482 but not in *i*CGB21FR were manually checked in MetaCyc (Caspi et al., 2020) or BioCyc (Karp et al., 2019) for their biochemical relevance in the model and, if appropriate, added. Altogether, 50 new reactions, 14 new metabolites, and 23 new genes were added to *i*CGB21FR. BiGG identifiers (IDs) and annotations were included in the model for all newly added compounds, thus enabling easier comparison with other models. If BiGG IDs were not yet existent, BioCyc IDs (Karp et al., 2019) and additional annotations such as SBO terms were added to the new instance.

#### 2.1.6. Mass and Charge Imbalances

The chemical formulas of all participating metabolites were verified. All mass and charge imbalanced reactions were manually checked. Pseudo-reactions, including exchange, sink, or biomass reactions, were excluded from this curation step. For reactions with imbalanced charge, the charge of every participating metabolite was verified and, if necessary, adapted. Mass imbalanced reactions were checked for missing metabolites, such as protons.

#### 2.1.7. Energy-Generating Cycles

Energy-generating cycles represent thermodynamically infeasible states. Charging of energy metabolites without any energy source causes such cycles (Fritzemeier et al., 2017). If left undetected in the model, these can result in erroneous increases in maximal yields in the biomass (Fritzemeier et al., 2017). The following 13 carrier metabolites for energy or redox equivalent were tested for their ability to form thermodynamically infeasible cycles: adenosine triphosphate (ATP), cytidine triphosphate (CTP), guanosine triphosphate (GTP), uridine triphosphate (UTP), inosine triphosphate (ITP), reduced nicotinamide adenine dinucleotide (NADH), reduced nicotinamide adenine dinucleotide phosphate (NADPH), flavin adenine mononucleotide (FMN), flavin adenine dinucleotide (FAD), menaquinol-8, 2-demethylmenaquinol 8, acetyl-CoA, and L-glutamate. All exchange reactions of the model were set to 0 mmol gDW^-1^ h^-1^ to investigate the presence of energy-generating cycles. Energy dissipating reactions were created for each of the 13 individual metabolites. These allow the corresponding metabolite to be removed from the system. Each reaction was added one-at-a-time to the model and then used as the objective function. If the optimization returned a result unequal to zero, an energy-generating cycle was detected and subsequently removed. Additionally, the proton exchange between cytosol and periplasm was included.

#### 2.1.8. Biomass Objective Function

The initial biomass objective function (BOF) of *i*CGB21FR was created using CarveMe (Machado et al., 2018). It represents a universal bacterial biomass objective function (BOF). The species-specific biomass objective function (BOF) was further refined using BOFdat (Lachance et al., 2019). BOFdat allows calculating and refining a pseudo-reaction for the biomass function without using any pseudo-metabolites or macromolecules, such as deoxyribonucleic acid (DNA), ribonucleic acid (RNA), or protein. The nucleotide sequence of *C. glutamicum* ATCC 13032 was used to refine the DNA nucleotides in the BOF. Coenzymes and inorganic ions were identified and specifically adapted for *C. glutamicum* in the BOF within the second step of BOFdat. As the model initially did not simulate growth on the minimal medium CGXII (see section 2.2.2), trace elements in the BOF were compared to the elemental composition of *C. glutamicum* cells (Liebl, 2005). Based on this comparison, cobalt was removed from the BOF.

#### 2.1.9. Subsystems and Groups Plugin

Biological pathways were obtained from the KEGG database (Kanehisa et al., 2019) using the old locus tags in the genes' annotations. Pathways associated with a reaction were added to the reaction's annotations based on genes in the GPR association. The pathways were added as a biological qualifier with the attribute OCCURS_IN. Additionally, the groups plugin was enabled, available for SBML Level 3. The groups plugin in libSBML (Bornstein et al., 2008) allows a more flexible grouping of specific connected components in the metabolic model (Hucka and Smith, 2016). The groups plugin was used to add every metabolic pathway or subsystem as a group. Participating reactions were then added to the groups as members.

#### 2.1.10. Quality Control

The quality of the GEM was tested performing a FROG analysis (König, 2020) and using Memote, version 0.11.1. Memote is a platform to test standardized measures of metabolic models and outputs quality scores ranging from 0 % for poor model quality to 100 % for excellent model quality (Lieven et al., 2020). The measures that generate the Memote scores evaluate the model's consistency and annotations within different categories. These categories include basic information about the model, the metabolites and reactions, the degree of annotations for metabolites, reactions, genes, and SBO terms. Memote also checks the presence of GPRs, a realistic biomass function, energy metabolism, and appropriate network topology. Apart from these individual Memote scores for the different subcategories, Memote also reports an overall score. This overall score represents an overall measurement of how well the model scored within all individual categories. To evaluate the consistency of the model, the stoichiometric consistency, mass and charge balances, metabolite connectivity, and unbounded fluxes in the default medium were used. Within the evaluation of the annotations, Memote checks for the presence and conformity of various databases and the presence of specific SBO terms. All categories are scored individually. The overall Memote quality score is calculated based on the individual category scores (Lieven et al., 2020).

#### 2.1.11. Curation of *i*CW773

The model *i*CW773 (Zhang et al., 2017) was downloaded in Microsoft Excel format as the supplementary published and converted to Character-Separated Value (CSV) format. The application Table2Model (Dräger, 2021) was developed based on JSBML (Rodriguez et al., 2015) to parse the CSV files and convert the information to SBML Level 3 Version 1 (Hucka et al., 2018). Since the original publication did not explicitly define any units, these had to be added to the model. For consistency reasons, the units were defined in the same way as for *i*CGB21FR. The generated SBML Level 3 Version 1 file was syntactically validated using a combination of JSBML (Rodriguez et al., 2015) and libSBML (Bornstein et al., 2008), including unit consistency validation. Memote version 0.11.1 (Lieven et al., 2020) was used for semantic model checking. Annotation of the model *i*CW773 was performed using the same curation pipeline described above with the help of ModelPolisher (Römer et al., 2016) and SBO term addition (Fritze, 2020). The model was wrapped in an (Bergmann et al., 2014) OMEX archive file (Neal et al., 2018) together with a metadata file and uploaded to BioModels Database (Malik-Sheriff et al., 2020), where it is available under accession MODEL2110010001 (see Availability).

### 2.2. Model Validation

All model validations were performed with a physiological pH of 7.0. The growth behavior was tested in several media with access to varying carbon sources under aerobic and anaerobic conditions to validate the predictive power of the curated model *i*CGB21FR.

#### 2.2.1. Definition of the Growth Unit

The growth rate is defined as the flux through the biomass objective function, which corresponds to the system's biomass-producing reaction. In their fundamental work from Varma and Palsson (1994) explain that “*V*_gro_ is the growth flux (grams of biomass produced), which with the basis of 1 g (dry weight) per h reduces to the growth rate (grams of biomass produced per gram [dry weight] per hour).” It should be noted that 1 gDW corresponds to 1 g with a semantic annotation regarding the dry weight fraction of a probe. Gottstein et al. (2016) explain that the metabolic fluxes are typically given in mmol gDW^-1^ h^-1^ and confirm (Varma and Palsson, 1994) that the growth rate μ has the unit g gDW^-1^ h^-1^. Gottstein et al. (2016) also state that the biomass objective function describes the accumulation of biomass components per hour and relative to the amount of biomass in gDW. Consequently, all molecular species need to be expressed in the unit mmol gDW^-1^, which corresponds to the amount of the biomass component per gram of biomass (cf. section 2.1; Gottstein et al., 2016). Since all stoichiometric coefficients have dimensionless units, the biomass forming reaction can be considered a summation of components in mmol gDW^-1^, each times a dimensionless factor. Consequently, the rate of this reaction, which defines a change per time, results in mmol gDW^-1^ h^-1^.

Accordingly, the SBML specification defines that the units of all reactions in a model have to be identical and are defined in units of *extent* per *time* (see Hucka et al., 2018, section 4.2.5; ). According to the specification of SBML Level 3 Version 1 Release 2 (see Hucka et al., 2018, Table 9), the extent units should be substance units or a combination of units derived from those. Here, the extent of the reactions and the substance units of all compounds are defined in units of mmol gDW^-1^ (note that in contrast to Varma and Palsson (1994), we here define the biomass in units of mmol instead of in g). The time units are defined in h (or 3600 s). Hence, all reactions have the unit mmol gDW^-1^ h^-1^. It should be noted that the upper and lower bounds of all reactions have the same unit and are therefore consistently defined with the flux through the biomass reaction. In this way, these parameters already implicitly define the flux units because the flux's upper and lower bounds must have the same unit as the flux itself.

For more information, readers may also consider the specification of the SBML extension package fbc (Olivier and Bergmann, 2018), which provides similar examples in its appendix, and the detailed analysis on this matter outlined by Gottstein et al. (2016). To improve the units' definition, *i*CGB21FR and *i*CW773 explicitly declare the attributes extentUnits and timeUnits within the model element in their SBML files. It also declares substanceUnits in mmol gDW^-1^ and the volumeUnits in fl so that all compounds and compartments inherit defined units from the model container.

Experimentally observed growth rates μ may be given in the unit 1/h. In this case, directly comparing the calculated growth rate to the experimentally obtained value is possible if the biomass consistency of a GEM approaches 1 mmol gDW^-1^ h^-1^ because then its produced biomass has a molecular weight of 1 g mmol^-1^. With this, the conversion 1 g gDW^-1^ h^-1^ = 1 g g^-1^ h^-1^ = 1 h^-1^ can be performed because the biomass of the GEM is, in this case, *standardized*. A direct comparison of growth rates is then valid, because with a biomass consistency close to 1 mmol gDW^-1^ h^-1^, the different units of the growth rate μ converge.

#### 2.2.2. Growth in Different Media and Conditions

Following common laboratory practice in cultivating *C. glutamicum*, the complete lysogeny broth (LB) medium (Bertani, 1951) and the two minimal media M9 (Sambrook et al., 1989) and CGXII (Keilhauer et al., 1993; Eggeling and Bott, 2005) were chosen to simulate *in silico* aerobic growth of *C. glutamicum*. Transporters for the inorganic ions nickel and calcium had to be added to allow growth on the M9 minimal medium. As protocatechuic acid is a component of the CGXII medium (Keilhauer et al., 1993), all necessary exchange and transport reactions were added to model the uptake this compound. The model *i*CGB21FR did initially not grow on the minimal medium CGXII. Literature research pointed toward cobalt in the BOF as a potential issue. Removing cobalt from the BOF allowed growth on CGXII.

D-glucose served as the predominant carbon source in the two minimal media. The composition of each medium was used to constrain the model's exchange reactions with the environment. For simulating growth in the three different media, the lower bounds of the metabolites' exchange reactions available in the respective medium were set to the default value -10 mmol gDW^-1^ h^-1^ to enable the uptake. All other exchange reactions' lower bounds were set to 0 mmol gDW^-1^ h^-1^. While applying these medium-specific constraints, the BOF was set as the objective function. If the model did not simulate growth on one of the experimentally confirmed media, literature was queried to identify missing metabolites or reactions hampering growth. These were then added to *i*CGB21FR.

*C. glutamicum* is a facultative anaerobe microbe (Eggeling and Bott, 2005, 440). The growth under anaerobic conditions was evaluated to demonstrate the validity of *i*CGB21FR. The model initially created with CarveMe (Machado et al., 2018) did not simulate growth when applying anaerobic conditions by blocking the oxygen uptake. The model was evaluated using flux balance analysis (FBA) to identify relevant oxygen-carrying reactions to identify potential reasons for this. Additionally, literature was searched to find alternative or missing reactions. Furthermore, the gap-filling option of CarveMe was used for the M9 minimal medium under anaerobic conditions. To this end, a novel draft model with CarveMe was created, where the gap-filling option was enabled during the curation step. The reaction set of the gap-filled model was compared to our extended *i*CGB21FR model's reaction set, and the missing reactions were added. These six missing reactions include the catalase reaction (CAT), the succinate dehydrogenase (SUCDi), the phosphoribosylformylglycinamidine synthase (PRFGS_1), a different calcium transporter (CAt4), the fumarate reductase (FRD7), and the glycolate transport via proton symport (GLYCLTt2rpp). With the inclusion of these reactions, the model simulated anaerobic growth on all three tested media.

The *C. glutamicum*-specific CGXII minimal medium was used to test the model's growth behavior on different carbon sources. The metabolites glucose, fructose, sucrose, ribose, gluconate, pyruvate, acetate, lactate, and propionate were tested under aerobic and anaerobic conditions as sole carbon sources since experimental data confirmed their role as carbon sources (Michel et al., 2015). All tested compounds could serve as the sole carbon source under aerobic conditions. Under anaerobic conditions, however, only glucose, fructose, sucrose, and ribose could serve as carbon and energy sources (Michel et al., 2015). Therefore, all nine carbon sources were tested *in silico* under aerobic and anaerobic conditions using the CGXII minimal medium. If *i*CGB21FR did not simulate growth on one of the experimentally verified carbon sources, missing exchange and transport reactions were added based on results from a literature search. These included adding a pyruvate exchange and transport reaction and a lactate transporter for the aerobic condition. Further gap-filling steps were performed when necessary.

#### 2.2.3. Verifying Capabilities for Amino Acid Production

The model was further validated by simulating the production of all 20 canonical amino acids in the CGXII medium and D-glucose as the predominant carbon source under aerobic conditions. The availability of D-glucose was restricted to the default uptake rate of 10 mmol gDW^-1^ h^-1^. The growth rate was fixed to 0.4 mmol gDW^-1^ h^-1^ to ensure the microbe's maintenance during the amino acid production. Subsequently, a sink reaction was created for each amino acid, set as the objective function, and optimized. The relative amino acid production was calculated by dividing the total amino acid production rate by the glucose uptake rate. The same approach was taken for the CO_2_ production rate, which was set in relationship to the amino acid production rate. The efflux of the CO_2_ exchange reaction (EX_co2_e) was taken as the CO_2_ production rate. The ATP production rate was calculated by summing up the fluxes of all ATP-producing reactions. These were then correlated with the amino acid production rate, analogously to the CO_2_ production rate.

### 2.3. Model Application: New Insights for Metabolic Engineering

As *C. glutamicum* is widely used in biotechnology, the model's capabilities can be used to yield hints for metabolic engineering. All subsequent analyses were performed using the CGXII minimal medium with D-glucose as the sole carbon source under aerobic conditions.

#### 2.3.1. Relation Between Growth and L-glutamate Production

A sink reaction (sink_glu__L) was added to optimize the L-glutamate production (see **Figure 5**). This reaction was then set as the objective function. As L-glutamate is also part of the biomass objective function, a potential association between these two reactions (BOF and L-glutamate sink reaction) was evaluated by varying the BOF between 0 and the maximum growth rate 0.57 mmol gDW^-1^ h^-1^ while maximizing the sink reaction. 0.57 mmol gDW^-1^ h^-1^ is the maximum *in silico* growth rate of *i*CGB21FR under aerobic growth conditions with D-glucose as the sole carbon source on CGXII (see section 3.2).

#### 2.3.2. Relevance of the pyruvate carboxylase (PC)

PC is a pivotal enzyme in the L-glutamate production in *C. glutamicum* (Peters-Wendisch et al., 2001). A metabolic map was drawn, which depicts the primary reactions relevant for the L-glutamate production starting at D-glucose as the predominant carbon source using the tool Escher (King et al., 2015, see **Figure 5**). The model was optimized for the sink reaction for L-glutamate (sink_glu__L) using FBA while fixing the growth rate to 0.4 mmol gDW^-1^ h^-1^. This growth rate is an intentionally chosen growth rate within the interval where the L-glutamate production is only marginally affected by growth. The resulting flux distribution is depicted in **Figure 6**. This analysis was repeated after knocking out the PC's reaction to elucidate further the PC's effect on the metabolic flux distribution.

#### 2.3.3. Identification of Relevant Reactions for L-glutamate Production

A loopless flux variability analysis (FVA) was used to identify reactions relevant to L-glutamate production. FVA represents a standard method to evaluate the range of feasible steady-state fluxes for each reaction by sequentially minimizing and maximizing each reaction (Schellenberger et al., 2011). Loop reactions are a subset of reactions with unbounded fluxes. Loopless FVA eliminates thermodynamically infeasible loops by not allowing the model to use these loops (Schellenberger et al., 2011). After running the loopless FVA, reactions with almost identical minimal and maximal allowed flux values were extracted (relative tolerance of 10^-5^, absolute tolerance of 10^-8^).

## 3. Results

### 3.1. The Model *i*CGB21FR Is of High Quality

The new GEM of *Corynebacterium glutamicum* constructed in this work is named *i*CGB21FR. This name follows the latest recommended naming conventions, which are part of the community standardization of metabolic models (Carey et al., 2020). The lower-case “*i*” in italics means *in silico*, followed by the species indicator “CG” for *C. glutamicum*. “B” represents the city where the particular strain ATCC 13032 was sequenced (Bielefeld, Germany, see also Kalinowski et al., 2003). The three-letter code “CGB” also serves the corresponding strain identifier in the KEGG pathway database (Kanehisa et al., 2019). It follows an iteration identifier, in this case, the year 21 of this century. The last two characters, “FR,” refer to the last names of the primary model curators.

The model *i*CGB21FR is available in the SBML Level 3 Version 1 format (Hucka et al., 2019) with the fbc plugin (Olivier and Bergmann, 2018) and the groups plugin (Hucka and Smith, 2016) enabled. It contains 1042 metabolites, 1539 reactions, and 805 genes. Thus, further 42 reactions, 13 metabolites, and 25 genes were added to the model following the initial draft reconstructed with CarveMe (Machado et al., 2018). All metabolites and reactions have a human-readable, descriptive name and a chemical formula. The model comprises the cytosolic, periplasmic, and extracellular compartments.

Its overall Memote score amounts to 87 %. The Memote score of the initial draft model created with CarveMe was 33 %. With intensive manual curation, the number of mass and charge imbalanced reactions could be diminished from an initial 170 to 19 imbalanced reactions. These represent 1.2 % of the total number of reactions. The model has a stoichiometric consistency of 99.7 % and does not contain any energy-generating cycles, dead-end metabolites, nor orphan metabolites.

Seventeen different databases are cross-referenced in the model's instances, yielding a Memote annotation score of 84 % for reactions, 84 % for metabolites, and 49 % for genes. Genes include cross-references to the three databases KEGG (Kanehisa et al., 2019), NCBI genes (Maglott et al., 2005), and NCBI proteins (Pruitt et al., 2005). Metabolites and reaction annotations contain cross-references to 13 and seven different databases, respectively. The databases BiGG (Norsigian et al., 2019), BioCyc (Karp et al., 2019), KEGG (Kanehisa et al., 2019), MetaNetX (Moretti et al., 2021), Reactome (Croft et al., 2010), and ModelSEED (Henry et al., 2010) are referenced for metabolites and reactions. Reactions also have cross-references to the RHEA database (Lombardot et al., 2019) and EC numbers. Metabolites have additional cross-references to the ChEBI database (Hastings et al., 2016), the Human Metabolome Database (HMDB) (Wishart et al., 2007), BioPath (Brandenburg et al., 2004), InChIKey (Heller et al., 2015), UniPathway (Morgat et al., 2011), lipid maps structure database (Sud et al., 2007), and the University of Minnesota Biocatalysis/Biodegradation Database (UM-BBD) (Ellis et al., 2003).

**Figure 2:**
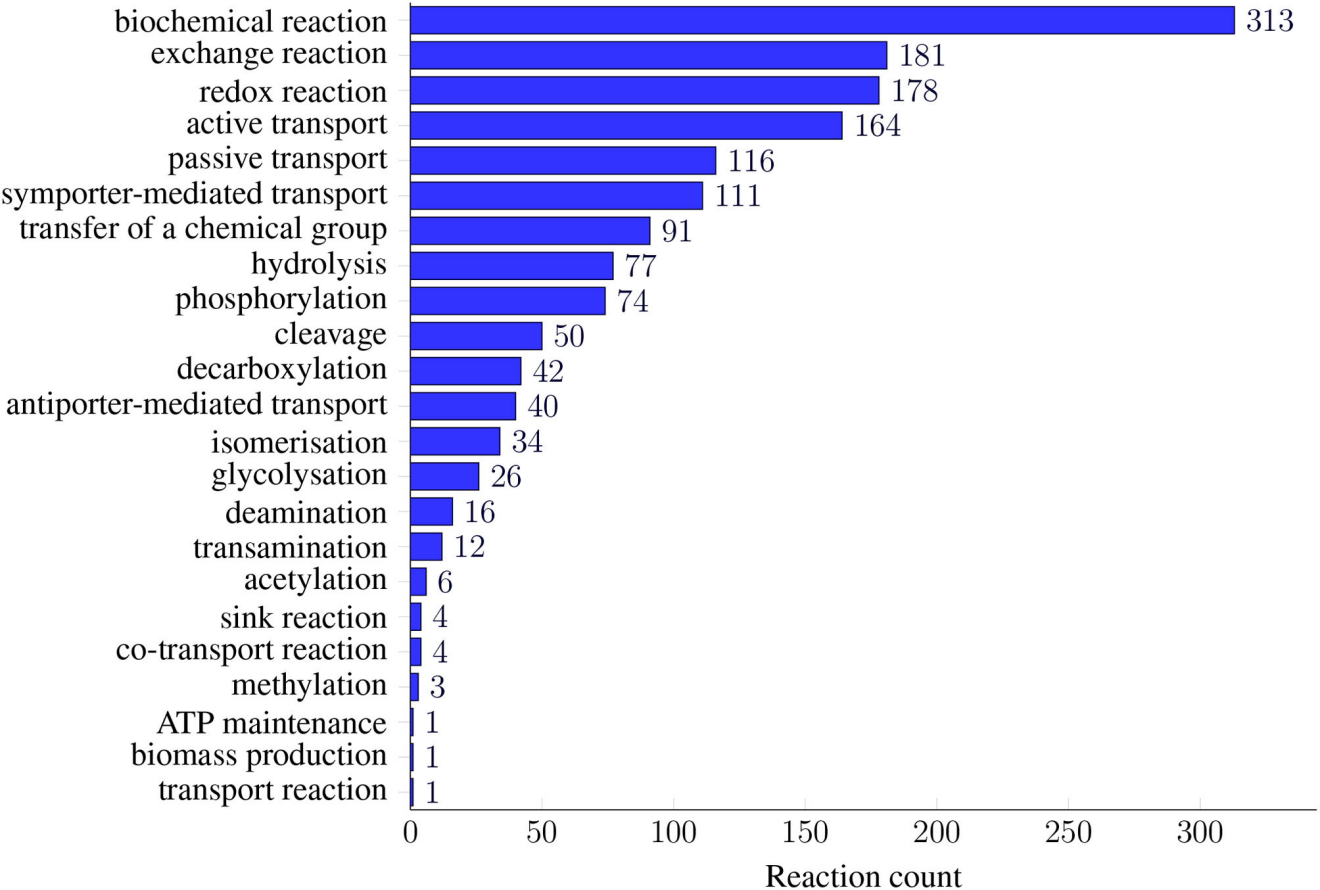
Prevalence of SBO terms in *i*CGB21FR. SBO terms for reactions were defined as precisely and specialized as possible. As many models only annotate their metabolic reactions with the most general SBO term for biochemical reactions (SBO:0000176), we further refined the SBO annotations. Thus, the model's reactions were annotated with 23 different SBO terms. They ranged from different transport reactions, including active, passive, or co-transport, to specific biochemical processes, like deamination, glycosylation, or isomerization. This fine-grained reaction annotation with SBO terms easily allows for subsequent analysis regarding reaction classes.

All model instances were further annotated using SBO terms (see Figure 2). While genes and metabolites received general SBO terms for genes and simple chemicals, the model's reactions were annotated with 23 different SBO terms. The most prominent ontology group is “biochemical reactions”: 313 reactions in the model hold the general SBO term for reactions. The number of biochemical reactions is followed by the group of exchange reactions with 181 reactions. The transport reactions are described more precisely by the SBO terms for active, passive, co-, symporter-mediated, antiporter-mediated, or general transport. For all other reactions, we identified SBO terms that describe the occurring biochemical reaction more precisely. In terms of ontology, these SBO terms are child nodes of the SBO term for biochemical reactions. These terms include, for example, redox reactions, the transfer of a chemical group, hydrolysis, or phosphorylation. The SBO terms for ATP maintenance and biomass production occur only once in the model. Figure 2 gives an overview of all 23 added reaction SBO terms and their occurrence in the model.

The plugins fbc (Olivier and Bergmann, 2018) and groups (Hucka and Smith, 2016) are enabled in *i*CGB21FR, thus allowing information such as metabolic charges, chemical formulas, or gene products to be stored. All identified KEGG pathways (Kanehisa et al., 2019) were added as a group to the model, with all reactions participating in the pathway as group members. In total, 102 groups were added to the model. The group with the most members is the “metabolic pathways” group with 563 members, followed by the “biosynthesis of secondary metabolites” group with 297 members. Other groups with more than 100 members are the “biosynthesis of amino acids” with 104 associated reactions, the “biosynthesis of cofactors” with 151 members, and the group of reactions associated with “microbial metabolism in diverse environments” with 171 members. With the help of these groups, reactions of a particular pathway can easily be extracted and analyzed.

The biomass objective function (BOF) created by CarveMe was refined in several steps to obtain realistic growth rates for the tested media. With the help of BOFdat and the nucleotide sequence of *C. glutamicum* ATCC 13032, the stoichiometric coefficients of the DNA nucleotides were adapted. The following seven metabolites were added as coenzymes and inorganic ions to the biomass objective function: NADH, NADPH, adenosine monophosphate (AMP), pyruvate, ammonium, sodium, and nickel. The stoichiometric coefficients of 16 further coenzymes and inorganic ions were adapted using BOFdat. The inorganic ion cobalt was removed from the BOF based on the elemental composition of *C. glutamicum* ATCC 13032 cells, as described by Eggeling and Bott (2005, p.16, 18). After including these changes, the simulated biomass production is in the range of a reasonable growth rate with no blocked biomass precursors in both the default and the complete medium.

### 3.2. Simulations of *i*CGB21FR Are Consistent With Experimental Data

**Figure 3:**
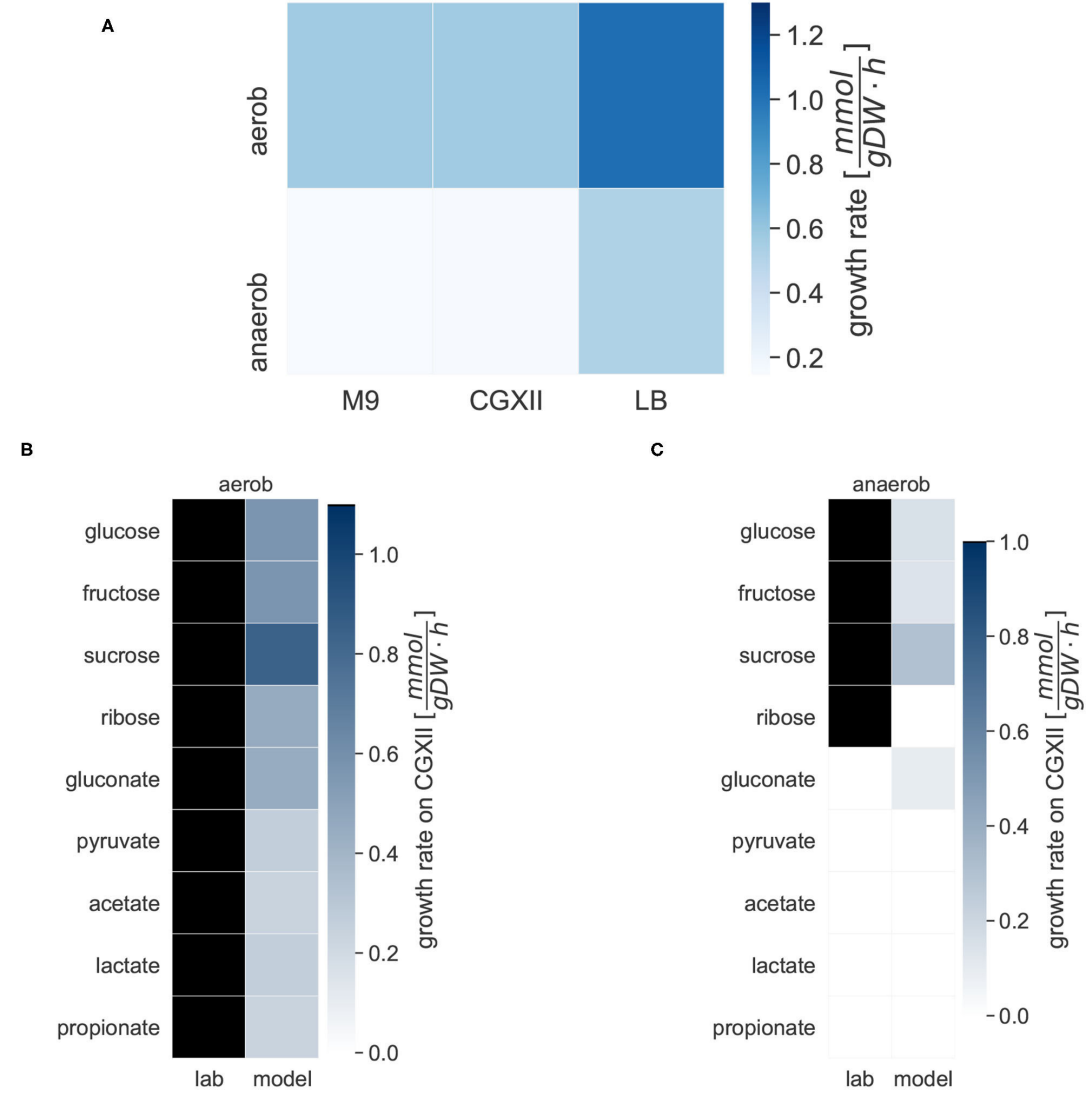
Specific growth rates of *C. glutamicum* under different conditions. Growth rates are given in mmol gDW^-1^ h^-1^. The darker the color, the higher the simulated growth rate under the given conditions. For the laboratory experiments, black indicates growth, and white indicates no growth on the given carbon source. **(A)** The *in silico* growth of *i*CGB21FR was simulated in the following three chemically defined media: the M9 minimal medium, the CGXII minimal medium, and the lysogeny broth (LB) complete medium. The growth was simulated under aerobic and anaerobic conditions. **(B)** Michel et al. tested the growth of *C. glutamicum* in CGXII minimal medium under aerobic conditions with different carbon sources in laboratory experiments. All tested compounds could serve as the sole carbon source under aerobic conditions (Michel et al., 2015). The different carbon sources were also evaluated with *i*CGB21FR. The model simulated growth on all given carbon sources. **(C)** The different carbon sources were also evaluated under anaerobic conditions. Michel et al. experimentally identified only glucose, pyruvate, sucrose, and ribose as carbon sources under anaerobic conditions. The model *i*CGB21FR was able to simulate growth on most of these sources as well except for ribose, but additionally showed growth on gluconate.

We simulated the growth of *i*CGB21FR in different media under aerobic and anaerobic conditions, and with access to different carbon sources (see Figure 3). Growth was tested on the two minimal media, M9 and CGXII, and the complete LB medium. The heat map in Figure 3A gives an overview of the growth behavior of *C. glutamicum* in the three different media under aerobic and anaerobic conditions. With 1.0266 mmol gDW^-1^ h^-1^ the biomass consistency of *i*CGB21FR is close to 1 mmol gDW^-1^ h^-1^. Consequently, it is approximately possible to directly compare the *in-silico* growth rate to an experimentally obtained growth rate given in 1 h^-1^. The simulated aerobic model growth on the minimal medium M9 with glucose as a single carbon source resulted in a maximal growth rate of 0.57 mmol gDW^-1^ h^-1^. A maximal realistic aerobic growth rate of 0.57 mmol gDW^-1^ h^-1^ on CGXII was obtained using the simulation tools COBRApy (Ebrahim et al., 2013) and confirmed with the Systems Biology Simulation Core Library (SBSCL) (Panchiwala et al., 2021). The simulated value is only slightly lower than the growth rate of 0.61 h^-1^ that Unthan et al. (2014) could experimentally obtain. The model simulates growth on the complex LB medium with a growth rate of 1.0214 mmol gDW^-1^ h^-1^ under aerobic conditions without further adjustments or refinements. As expected, the growth rate in the complex medium (LB) is approximately twice as high as in the two minimal media (M9 and CGXII). The aerobic growth conditions in all three media show a higher simulated growth rate compared to the anaerobic conditions, as anticipated. All growth rates are within a realistic range (Unthan et al., 2014).

The growth of *C. glutamicum* in CGXII minimal medium under aerobic and anaerobic conditions with varying carbon sources was tested. Under aerobic conditions, the model simulated biomass production on all carbon sources. Aerobic growth was possible on all carbon sources. The growth rates varied between 0.8437 mmol gDW^-1^ h^-1^ on sucrose and 0.2401 mmol gDW^-1^ h^-1^ on acetate. In our anaerobic *in silico* experiments, biomass production was also possible on three of the experimentally validated carbon sources, but not on ribose. Additionally, gluconate can be used as carbon sources. The biomass production on gluconate yielded a rate of 0.0945 mmol gDW^-1^ h^-1^.

**Figure 4:**
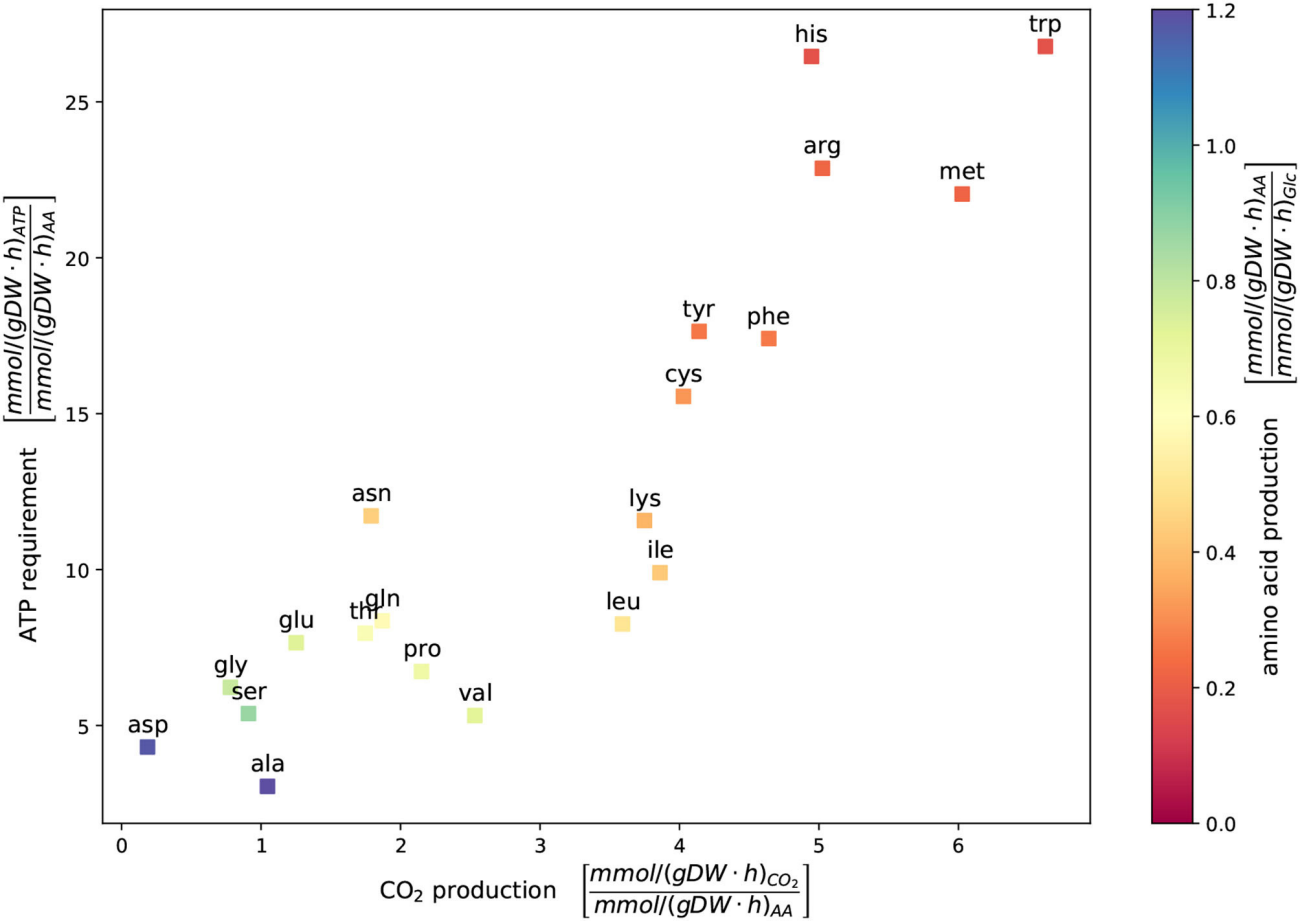
Metabolic cost of amino acid production. The ATP requirement depicted vs. CO_2_ production and the *in silico* amino acid production were determined using flux balance analysis (FBA). Glucose served as a sole carbon source under aerobic growth conditions. The availability of glucose was restricted by an uptake rate of 10 mmol gDW^-1^ h^-1^. The growth rate was fixed to 0.4 mmol gDW^-1^ h^-1^ to ensure the organisms' viability. Each square represents the result of a single FBA with the objective to maximize the corresponding amino acid production. The color indicates the amino acid production rate in relationship to the glucose uptake rate. In this simulation, l-aspartate and l-alanine, result in the highest amino acid production rate with 11.77 mmol gDW^-1^ h^-1^ and 13.73 mmol gDW^-1^ h^-1^, respectively. A trade-off exists between the yield of amino acid production and the energy expenditure and CO_2_ production: the more ATP is required, the more CO_2_ and lower amino acid production rates are yielded.

The new model of *C. glutamicum* can simulate the production of all 20 canonical amino acids while growing on the CGXII medium with D-glucose as the carbon source under aerobic conditions. In Figure 4, each square represents the result of a single FBA with the objective to maximize the corresponding amino acid production. The color indicates the amino acid production rate with respect to the glucose uptake rate. The positioning represents the ATP requirements and CO_2_ production in relation to the amino acid production rate. The two amino acids, l-aspartate (asp) and l-alanine (ala), have the highest absolute amino acid production rates with 11.77 mmol gDW^-1^ h^-1^ and 13.73 mmol gDW^-1^ h^-1^, respectively (see also Zelle et al., 2015). In contrast, the amino acids l-histidine, l-arginine, and l-tryptophan have the lowest amino acid production rate and the highest ATP requirements. A relationship exists between the yield of amino acid production, energy expenditure, and CO_2_ production: The more ATP is required, the more CO_2_ produced and the lower is the amino acid production rate. L-glutamate is of particular interest for metabolic engineering in *C. glutamicum*. Its total production rate under the selected conditions yields 8.7 mmol gDW^-1^ h^-1^.

### 3.3. Pointers to Metabolic Engineering for the L-glutamate Production

**Figure 5:**
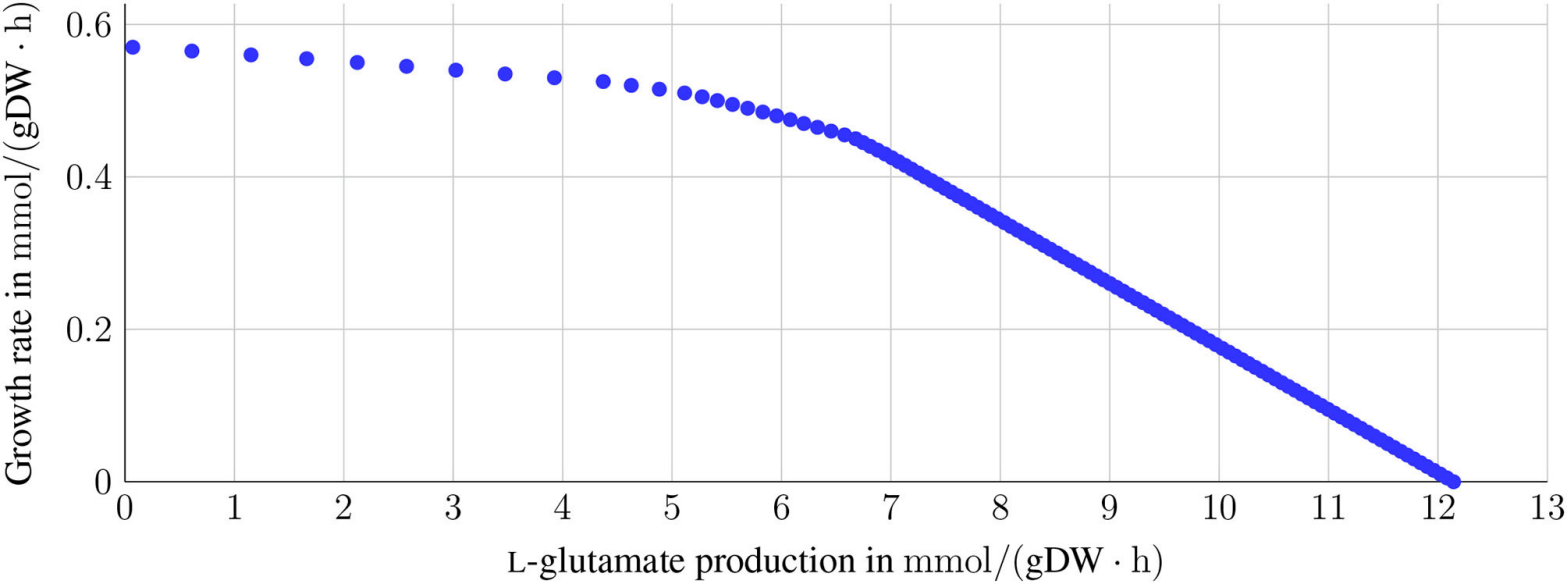
Trade-off between L-glutamate production and growth. The sink reaction for L-glutamate was set as the objective function to investigate the relation between the production of L-glutamate and growth. The growth rate was varied between 0 and the maximum growth rate of 0.57 mmol gDW^-1^ h^-1^ with glucose as the sole carbon source on CGXII. A dependency between growth and L-glutamate production is expected, as L-glutamate is part of the growth function with a stoichiometric coefficient of 0.0149. For growth rates between 0 and up to 0.4 mmol gDW^-1^ h^-1^, the L-glutamate production only decreases slightly (from 12 to approximately 7 mmol gDW^-1^ h^-1^. In contrast, the production of L-glutamate decreases drastically for higher growth rates.

*C. glutamicum* is a well-known L-glutamate producer. However, L-glutamate is also required for the growth or maintenance function. L-glutamate accounts for the growth function with a stoichiometric coefficient of 0.0149. Thus, a trade-off between growth requirement and the production of L-glutamate is expected. This trade-off is depicted in Figure 5. For growth rates between 0 mmol gDW^-1^ h^-1^ and 0.4 mmol gDW^-1^ h^-1^, the L-glutamate production rate remains comparably high. It only decreases by 5 mmol gDW^-1^ h^-1^. With increasing growth rates greater than 0.4 mmol gDW^-1^ h^-1^, the L-glutamate production rate decreases rapidly.

**Figure 6:**
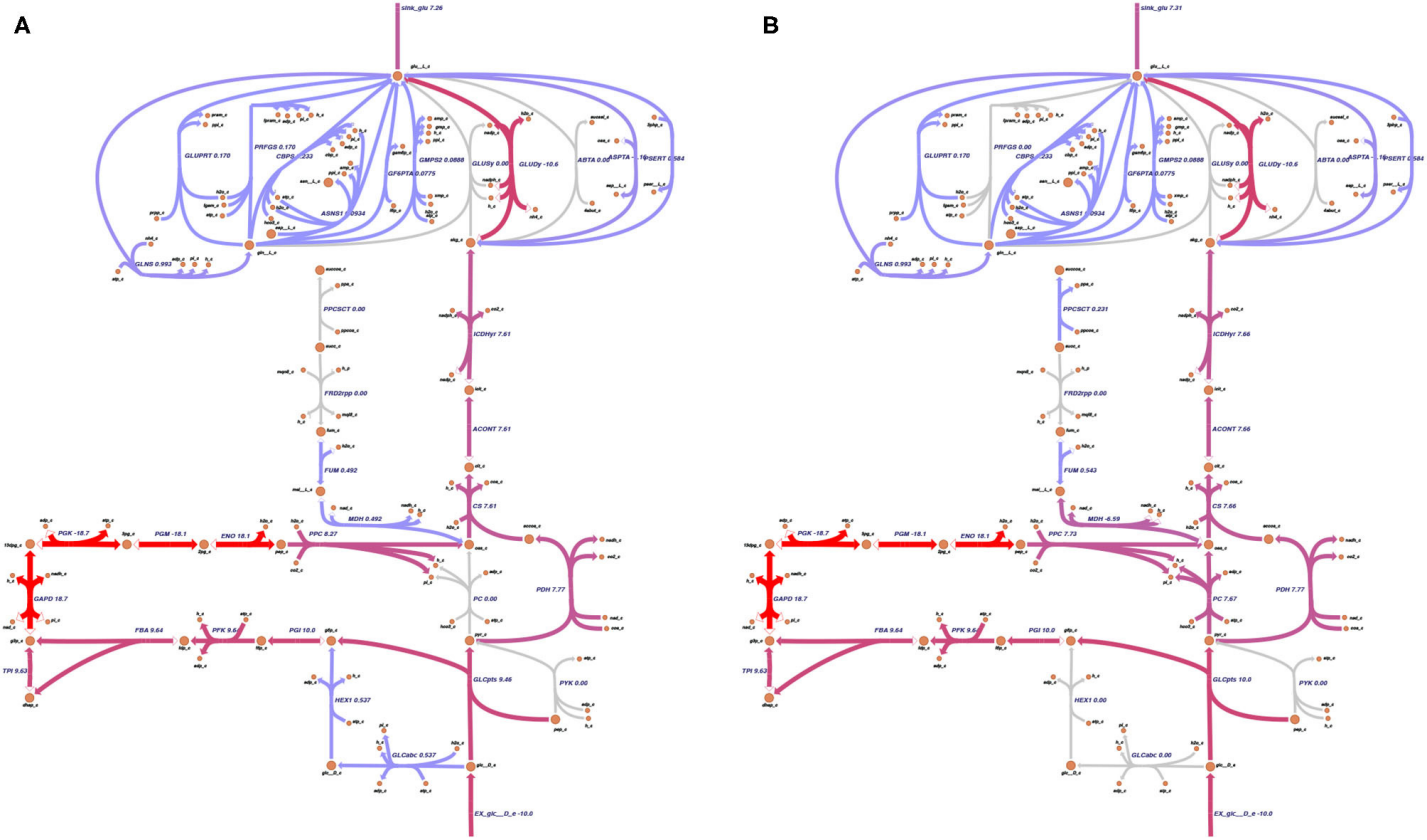
Pyruvate carboxylase and the glutamate production. As the pyruvate carboxylase (PC) was discovered to be the bottleneck of glutamate production (Peters-Wendisch et al., 2001), its knock-out effect on the metabolic model was analyzed. For the simulation, the growth rate was fixed to 0.4 mmol gDW^-1^ h^-1^, and the glutamate production was set as the objective function. The PC reaction was knocked out. The predicted flux distribution under maximal L-glutamate production resulting from the flux balance analysis (FBA) was plotted on the metabolic map, which was drawn using Escher (King et al., 2015). **(A)** shows the predicted flux distribution of the knock-out of the PC under maximal L-glutamate production. **(B)** depicts the predicted flux distribution of the wild-type model. In both cases, L-glutamate is produced. The maximal glutamate production rate only decreases by 0.05 mmol gDW^-1^ h^-1^ when the PC is knocked out. Thus, the model *i*CGB21FR can compensate for the loss of the PC reaction.

The PC plays a pivotal role in L-glutamate production (Peters-Wendisch et al., 2001). The effect of a knock-out of the PC on the flux distribution is depicted in Figure 6. Knocking out the PC decreases the L-glutamate production only to a small extent (from 7.31 mmol gDW^-1^ h^-1^ to 7.26 mmol gDW^-1^ h^-1^). The limiting factor in L-glutamate production is the availability of a carbon source (in this example, D-glucose) and, as shown above, the growth rate. The PC *in silico* knock-out experiment indicates that *C. glutamicum* can compensate for the knocked-out reaction.

Performing FVA helps to identify potential the ranges of each flux. Reactions relevant for optimizing the objective function can be identified by filtering for reactions with almost identical minimal and maximal flux values. With loopless FVA, we identified six highly relevant reactions for L-glutamate production in *C. glutamicum*. Among these six reactions were two pseudo-reactions: the exchange reaction of D-glucose and the sink reaction for L-glutamate. Glucose is the sole carbon source in the *in silico* experiment. Therefore, its strong influence on L-glutamate production is apparent. The same holds for the sink reaction that was used as the objective function in the FVA. The other four relevant reactions include the aconitate hydratase (ACONT), which converts citrate to isocitrate, the citrate synthase (CS), which converts acetyl-CoA and oxaloacetate to citrate and coenzyme A, the glucose transport via phosphoenolpyruvate, and the isocitrate dehydrogenase (ICDHyr), which converts isocitrate to 2-oxoglutarate (see also Table 1). The reactions ACONT, CS, and ICDHyr represent the fragile connection between glycolysis and L-glutamate biosynthesis. This connection can additionally be seen in Figure 6, where the three mentioned reactions are also illustrated.

**Table 1:** Reactions of particular relevance for the production of L-glutamate in *C. glutamicum*.

**Reaction ID**	**Reaction name**	**Reaction**
ACONT	aconitate hydratase	Citrate ⇌ Isocitrate
CS	citrate synthase	Acetyl-CoA + H_2_O + Oxaloacetate → Citrate + Coenzyme A + H^+^
EX_glc__D_e	D-glucose exchange	D-Glucose ⇌∅
GLCpts	D-glucose transport via PEP:Pyr	D-Glucose + Phosphoenolpyruvate → D-Glucose 6-phosphate + Pyruvate
ICDHyr	isocitrate dehydrogenase (NADP)	Isocitrate + NADP ⇌ 2-Oxoglutarate + CO_2_ + NADPH
sink_glu__L	sink reaction for L-glutamate	L-glutamate → ∅

*Reactions identified by flux variability analysis (FVA) to be highly relevant for L-glutamate production*.

## 4. Discussion

An updated genome-scale metabolic model *i*CGB21FR of *C. glutamicum* ATCC 13032 was reconstructed and validated using newly available specialized reconstruction tools. Using recent tools, the phenotypic prediction of the model's metabolism allows a more accurate depiction of the metabolic capabilities of *C. glutamicum*. This GEM was created using current community standards for high-quality reconstructions. The new *in silico* model reproduces experimentally validated data. In addition, we also curated *i*CW773 to meet systems biology standards (see also Carey et al., 2020). Initially, the model *i*CW773 was only available as a spreadsheet in Microsoft Excel format. According to the profound debate within the systems biology community (see Ebrahim et al., 2015), using this format is no longer recommended because it does not support unambiguous interpretation and direct reuse in further model analysis, especially for non-computational scientists. Generally, models in spreadsheet files do not fully support the principles of findable, accessible, interoperable, reusable data in science (Wilkinson et al., 2016) because using them in computational analyses requires converting these files to a standardized format. After converting *i*CW773 to SBML Level 3 Version 1 and performing several curation steps, it now contains SBO terms and has a Memote score that was increased from initially 29 % to 70 %. However, precaution is advised when using *i*CW773 as it contains reconstruction inconsistencies and incorrect metabolites. The curated *i*CW773 is available in SBML Level 3 Version 1 on the BioModels Database (Malik-Sheriff et al., 2020) under the accession number MODEL2110010001 (see Availability below).

### 4.1. Reconstruction Is of High Quality

The comprehensive annotations of all model components, including metabolites, reactions, and genes, contribute to the high quality of the reconstruction of *i*CGB21FR. Each instance is uniquely referenced to at least one database, thus providing a permanent link to clearly and uniquely identify this instance with its attributes (Juty et al., 2012). Almost all model instances are annotated with references to a minimum of one database, allowing more precise cross-referencing and interoperability between different databases. This high level of annotations is advantageous, as findable, accessible, interoperable, reusable (FAIR) data principles allow fellow scientists to conduct research on and with this model continuously (Dräger and Palsson, 2014; Wilkinson et al., 2016). Erroneous or missing information, incompatible data formats, or missing annotations significantly hamper the reuse of GEMs (Ravikrishnan and Raman, 2015). Missing annotations can lead to identification problems of compounds and reactions. GPRs are added for different reactions, and all instances are equipped with SBO terms, facilitating FAIR data principles.

Generally, the high degree of annotations in *i*CGB21FR is confirmed by the high Memote scores within the different categories. In terms of the presence of annotations, almost all Memote scores of the annotation categories rank close to 100 %. This high score implies that almost all suggested standards concerning annotations are met for this GEM (see also Carey et al., 2020). The current version of Memote does not include all *C. glutamicum*-specific databases, while other organism-specific databases with less relevance for our model are incorporated. One example can be found in the annotations section of the genes, where cross-references to different *Escherichia coli* databases are checked. In *i*CGB21FR, the Memote score for the presence of SBO terms for biochemical reactions sticks out due to its comparatively low value. The current version of Memote checks every reaction for the annotation with the most general SBO term (SBO:0000176), “biochemical reaction.” This check implies that Memote can not yet capture the fine-grained description of biochemical reactions in this model. Thus, the score of the metabolic reactions of 33.4 % diminishes the overall Memote SBO term annotation score.

Two typical ways exist to calculate the biomass objective function (BOF) of an organism. These are the macromolecular-based and the sequence-based approach. A typical biomass objective function (BOF) comprises the cell's primary macromolecules, essential coenzymes, inorganic ions, and species-specific metabolites, including the cell wall components. Additionally, the energy requirements for growth and non-growth associated maintenance costs are included (Lachance et al., 2019). Using an experimentally derived biomass composition implies that its cellular composition depends on the experimental conditions under which it was obtained. For example, the availability of nutrients and the resulting growth rate influence the ratio between DNA, RNA, and proteins (Scott et al., 2010). It thus represents a biased approach to compute the biomass. When no species-specific experimental data for the sequence-based approach is available to calculate a species-specific biomass function, a universal bacterial biomass function is included. Adapted biomass composition of a highly developed and curated GEM of *E. coli* is often used (Orth et al., 2011; Xavier et al., 2017). The new model possesses a biomass function adapted specifically to *C. glutamicum*. This conceptual approach differs from previous works by Kjeldsen and Nielsen (2009) and Zelle et al. (2015). For the BOF of Kjeldsen and Nielsen (2009), a biomass equation and the corresponding energy consumption associated with each reaction were formulated for each macromolecule. No *C. glutamicum*-specific data for the energy requirement of the polymerization of macromolecules was available; thus, *E. coli* data was used instead. The BOF of *i*EZ482 is based on the BOF of Kjeldsen and Nielsen (2009). Using species-specific data forms the basis for models with high predictive value. BOFdat enables the curation and refinement of a species-specific BOF by incorporating various -omics data into its calculation. In this study, genomics data were available and applied to refine the species-specific BOF.

### 4.2. *i*CGB21FR Reproduces Experimentally Obtained Data

The model *i*CGB21FR was validated by simulating growth on three different media under aerobic and anaerobic conditions. *C. glutamicum* is also known to grow in the brain heart infusion (BHI) medium. Modeling requires chemically defined media for growth simulations. We could not test the growth of *i*CGB21FR in BHI as no exact composition of the chemical definition of this medium exists. Simulating aerobic growth on LB complete medium was possible without any additional refinement of the model. Aerobic growth on the two minimal media was only possible by adapting the biomass function for growth on CGXII and adding missing reactions to the model for growth on M9. Missing reactions were identified by literature research. Anaerobic growth was enabled after adding six reactions. By this, the model was refined step by step to simulate and confirm already known growth conditions.

*In silico* growth rates were higher on the complete medium compared to the two minimal media. The aerobic growth rates were higher than the anaerobic growth rates, with all growth rates within a realistic range (Unthan et al., 2014; Michel et al., 2015). Both findings are expected, as complete media provide more nutrients and biomass precursors than minimal media. As their name suggest, minimal media only provide minimal required nutrients for the organism to grow. *C. glutamicum* uses oxygen and the more efficient aerobic respiration. It is even often regarded as aerobe (Takeno et al., 2007). However, as *C. glutamicum* is facultatively anaerobe, it can also switch to fermentation and anaerobic respiration if oxygen is absent. Anaerobic growth by nitrate respiration is limited, as nitrate accumulates and inhibits growth. Additionally, glucose is converted to l-lactate and succinate without the growth of the organism (Inui et al., 2004b; Koch-Koerfges et al., 2013).

Two observations stick out from these growth results. First, the current tools for the reconstruction of GEMs still demand subsequent manual refinement. Even though automated tools, such as CarveMe (Machado et al., 2018), reduce the amount of time spent on the reconstruction dramatically, manual refinement remains a pivotal part of the reconstruction process. The necessity of manual curation becomes particularly apparent when comparing the growth predictions of our model for the different media. The draft model created by CarveMe enabled growth on the complete medium without further ado. However, manual refinement was essential for the simulation of growth on the two minimal media. The second interesting observation is the organism-specific gap-filling, which appears to be more fruitful when applied to media that specifically compensate for certain physiological or metabolic oddities of the organism. In our case, knowledge gap-filling was most fruitful on the CGXII medium which, for example, compensates for the limited ability of *C. glutamicum* to synthesize and excrete siderophores (Budzikiewicz et al., 1997). This makes sense, as the minimal medium provides the microbe's bare necessities to grow. Potentially lacking compounds could be compensated by the composition of the complete medium.

We validated our model by testing the growth rate on the CGXII minimal medium under aerobic and anaerobic conditions using different experimentally validated carbon sources. Aerobic growth was possible on all experimentally validated carbon sources. In addition to the experimentally confirmed anaerobic growth on glucose, fructose, sucrose as carbon sources, our *in silico* model also grew on gluconate. We verified that all genes required to utilize gluconate as carbon sources exist in *C. glutamicum*. The rate of NADPH reoxidation could be a potential explanation for the *in silico* growth on the additional anaerobic carbon source. The enzyme 6-phosphogluconate dehydrogenase oxidizes 6-phosphogluconate to ribulose 5-phosphate. This enzyme is inhibited by NADPH, which is essential for the cellular control of the NADPH synthesis (Moritz et al., 2000). The rate of NADPH re-oxidation represents a critical element of this process. Gluconate is phosphorylated after uptake and then catabolized in the pentose phosphate pathway. If NADPH re-oxidation was too low under anaerobic conditions, NADPH could accumulate and result in complete inhibition of 6-phosphogluconate dehydrogenase activity. This accumulation would lead to a stop in growth on gluconate, as was shown by experimental data (Michel et al., 2015). If, however, NADPH re-oxidation is sufficiently fast and no NADPH accumulates, the activity of the 6-phosphogluconate dehydrogenase could remain active and allow anaerobic growth on gluconate by simulation studies with this *in silico* model.

As a final validation step of the metabolic model, its ability to produce amino acids was examined. In complex bacterial systems, amino acid production co-occurs with growth (Marx et al., 1996). The biosynthesis of amino acids requires a lot of the carbon source's total budget, usually used for bacterial growth (Neidhardt et al., 1990). The growth rate was fixed to 0.4 mmol gDW^-1^ h^-1^ to ensure the microbe's viability while producing amino acids. With increasing CO_2_ production rate and ATP requirements, the amino acid production yield decreases. Especially smaller amino acids with only a few carbon atoms, like l-alanine with only three carbon atoms, or glycine with two carbon atoms, have a low CO_2_ production and ATP requirement rate.

In contrast, amino acids with more carbon atoms, such as l-tryptophan, have a much higher ATP requirement and CO_2_ production rate, while the amino acid yield is relatively low. This leads to the conclusion that building more extensive and more complex amino acids needs more energy. An increasing proportion of the consumed sugar will be used to produce more energy, is then lost as CO_2_, and cannot be used for amino acid formation (Gourdon et al., 2003).

### 4.3. L-glutamate Production: New Insights for Metabolic Engineering

One might expect a linear correlation between the L-glutamate production and the growth rate, where the slope is related to the amino acid's stoichiometric coefficient in the biomass function. The trade-off between the production of L-glutamate and the growth rate was investigated by fixing the growth rates and maximizing the L-glutamate production (see Figure 5). The system moves between these two boundaries: the maximal possible growth rate and the maximal possible production of L-glutamate. The closer the values get to either maximum, the greater is the influence on the respective other value. When ceasing growth, the theoretical production of L-glutamate would reach a maximum since the available metabolic capacity is invested in the L-glutamate production. The reverse situation occurs with ceasing L-glutamate production and maximizing growth where all energetic demand is invested. L-glutamate production reaches its maximum when no growth occurs, and the available glucose is completely used to produce L-glutamate. Thus, the growth rate is the limiting factor for our *in silico* model, independent of the L-glutamate production.

The PC was first investigated to study relevant reactions for L-glutamate production (see Figure 6). The PC has been described as the bottleneck in the production of L-glutamate (Peters-Wendisch et al., 2001). Knocking out the PC in laboratory experiments leads to ambivalent results: Both drastic decrease (Peters-Wendisch et al., 2001) and increase (Sato et al., 2008) in L-glutamate production were reported after a disruption of the PC. Pyruvate is part of the complex network responsible for carboxylation and decarboxylation reactions, which connect the glycolysis and TCA cycle (Becker and Wittmann, 2020). In *C. glutamicum*, the PC represents one of the carboxylation enzymes, the other being the phosphoenolpyruvate carboxylase (Eikmanns, 2005). The carboxylation and decarboxylation enzymatic complex in *C. glutamicum* is a highly flexible network that enables several pathways to respond to varying metabolic circumstances (Möllney et al., 2000; Becker et al., 2008; Becker and Wittmann, 2020)—knocking out the PC in our model still allowed L-glutamate production. We also found that the amount of produced L-glutamate does not vary significantly with the PC being knocked. According to our simulation, the limiting factors in L-glutamate production are access to carbon sources and the growth rate.

Four reactions were identified that play a pivotal role in the production of L-glutamate in our model. The first is a glucose transporter, which uses the phosphoenolpyruvate (PEP)-dependent sugar phosphotransferase system. The L-glutamate yield decreased with sugar consumption rates in laboratory experiments (Gourdon et al., 2003). This seems reasonable, as glucose is the sole carbon source and starting point for glutamate production. Increasing glucose availability is only expedient if the glucose transporters' capacity is given to take up the enhanced supply of glucose. The three remaining reactions, aconitase, citrate synthase, and isocitrate dehydrogenase, are all part of the tricarboxylic acid (TCA) cycle. The TCA cycle is a complex regulated amphibolic pathway with L-glutamate and l-lysine as derived intermediate products (Bott, 2007).

The aconitase gene is regulated by four transcriptional regulators, indicating a tight control of this enzyme. A *C. glutamicum* mutant lacking the aconitase gene was glutamate auxotrophic in the CGXII minimal medium with glucose as the carbon source (Baumgart et al., 2011). The model *i*CGB21FR could replicate the finding that aconitase is essential for L-glutamate production. It remains to be further experimentally validated how the interplay between the aconitase within the TCA cycle in terms of L-glutamate production can be optimized.

The citrate synthase catalyzes the initial reaction of the TCA cycle. Overexpression of the citrate synthase can redirect more carbon flux into the cycle and result in higher l-arginine production (Man et al., 2016). l-arginine is synthesized from the precursor l-glutamic acid (Utagawa, 2004). Thus, higher production of L-glutamate might also be dependent upon the activity of the citrate synthase. The role of the citrate synthase in L-glutamate production might be an interesting topic to investigate since it might represent a target for metabolic engineering of *C. glutamicum*'s TCA cycle. Since the citrate synthase is the initial reaction of the TCA cycle with L-glutamate and l-lysine as intermediates, its activity might prove particularly fruitful.

Isocitrate dehydrogenase catalyzes the oxidative decarboxylation of isocitrate. Becker et al. (2009) found in their investigation of the effects of the isocitrate dehydrogenase on l-lysine production that decreased activity of the isocitrate dehydrogenase improves the l-lysine production. This decrease induced a flux shift from the TCA cycle to anaplerotic carboxylation (van Ooyen et al., 2012). However, the PC functions as an anaplerotic enzyme in L-glutamate production (Peters-Wendisch et al., 1997). In other words, the isocitrate dehydrogenase has different functions in L-glutamate production than in l-lysine production. This differing function becomes more apparent when looking at the effects of isocitrate dehydrogenase inactivation in *C. glutamicum*: Inactivation of the NADP-dependent isocitrate dehydrogenase in *C. glutamicum* leads to L-glutamate auxotrophy (Eikmanns et al., 1995). This connection between the PC and the isocitrate dehydrogenase in L-glutamate production might be an interesting target for metabolic engineering.

## 5. Conclusion and Outlook

The new model *i*CGB21FR represents an GEM of high quality of the biotechnologically relevant microorganism *Corynebacterium glutamicum* ATCC 13032. We reconstructed this metabolic model with an adapted, species-specific biomass composition and realistic growth rates in different environments, which were validated using experimentally derived data. Furthermore, alternative metabolic pathways for the production of L-glutamate were shown in our *in silico* model. Particularly, these alternative pathways could be of interest for further investigation in terms of metabolic engineering. Biotin is a key player for the L-glutamate production in *C. glutamicum* since its limitation triggers L-glutamate production. Despite the inclusion of biotin in *i*CGB21FR and its participation in five biochemical reactions, its role in L-glutamate production is currently not included. The influence of biotin on the PC and the reactions involved in the alternative pathway for L-glutamate production with the pyruvate carboxylase knocked-out should be further investigated in subsequent GEMs of *C. glutamicum*.

## Data Availability Statement

The datasets presented in this study can be found in online repositories. The names of the repository/repositories and accession number(s) can be found in the article/Supplementary Material.

## Author Contributions

MF and AR curated and refined the model and conducted the study. MF, AR, and AD wrote the manuscript. EZ, KN, and WW revised the manuscript. AD supervised the study. All authors reviewed and approved the final manuscript.

## Funding

The authors acknowledge support by the Open Access Publishing Fund of the University of Tübingen (https://uni-tuebingen.de/en/58988).

## Conflict of Interest

The authors declare that the research was conducted in the absence of any commercial or financial relationships that could be construed as a potential conflict of interest.

## Publisher's Note

All claims expressed in this article are solely those of the authors and do not necessarily represent those of their affiliated organizations, or those of the publisher, the editors and the reviewers. Any product that may be evaluated in this article, or claim that may be made by its manufacturer, is not guaranteed or endorsed by the publisher.
